# Extracting Teeth

**Published:** 1866-05

**Authors:** A. S. Talbert

**Affiliations:** Lexington, Ky.


### EXTRACTING TEETH.

A. S. TALBERT, D. D. S., LEXINGTON, KY.

(Read before the Mississippi Valley Association of Dental Surgeons, at their annual meeting, February 23, 1866

GENTLEMEN OF THE ASSOCIATION:—Having been appointed at our last meeting to "speak a peace" at this, I shall feel that my task is accomplished, if I suceed in *entertaining* you a few minutes, though possibly from my age and experience, some of the younger members might expect to be instructed, as well as entertained. I know "but little," nor that little much.

If I make a long journey from home, I do not meet with *very many*, nor very startling incidents! nor yet do I make the acquaintance of either "confidence" men or women! If I go fishing, I do not catch very *many*, nor very *large fish*; and I may add, I do not "*take a smile*" often enough to constitute me an "*active* member" of the piscatorial fraternity!

If at home I read my Bible, and meditate on the visions of the Prophets, I do not see their fulfillment in the passing events of my life, nor do I see the dawn of the Millenium, lighting up the portals of the near future, I do not hear the

"music of the spheres," nor do I commune with the *spirits of the departed.* If in my office, by dint of hard labor, I sometimes suceed in making a good operation, I do not think it very wonderful. But if, of the little I do know, I can add one mite to the interest of this association, or to the advancement of my profession, I cheerfully make the contribution.

Much has been said, and much has been written, on the subject of extracting teeth. More might be said; for, as the fifty-two cards of a deck, may be shuffled, an indefinite number of times, and each cut show a new trump, so of the fifty-two teeth, liable at different periods of life to need extraction, each presents a new feature in each individual for whom we operate. More judgement is often required in diagnosis than skill in operating, the healing power of nature will soon hide the blunders of the unskillful; but it will not restore a lost tooth.

No unvarying rules, however, can be given, defining the exact time when either deciduous or permanent teeth ought to be extracted. The age of the patient, condition of the tooth itself, its relation to those around it, the general health of the patient, its local or general effect upon the health, or upon the appearance of the patient, all these, and many other considerations are to bear on the question of extracting, or preserving any given tooth. Perfection of development no doubt depends on perfect health; but apparently perfect health does not always produce perfection of development in the teeth.

I shall not attempt to show when a tooth should be extracted, but content myself with a few words, as to the manner of doing it, after it has been decided to be necessary. It is fair to presume that you all understand the relations which the teeth sustain to the surrounding parts; each tooth being firmly held in its socket by means of a ligament, the rupture of which must necessarily occur in its extraction. Physical force, if applied with persistent energy, is alone sufficient to break up this connection; but as you would not set a board edgewise to break it, so you would more easily break this ligamentous attachment, by lateral rotary, internal, and external motion, combined with direct force.

But little preliminary operating is usually required, for their attachments are so protected by the alveolor processes that the knife will not reach them, except merely around their borders. Hence it is not often necessary to "lance the gums," which will not be lacerated by the operation, unless the process itself be broken, and come away with the tooth; and no human forsight can divine whether or not this will be the result. Since my earliest experience I have seldom used the lancet. In the use of anæsthetics, I found it impracticable, and that led me to see that it can generally be dispensed with; and as all patients contemplate extracting with horror, I endeavor to assuage their fear as much as possible, by presenting but few preliminaries, making but little display of instruments, and, without delay, make *one* instead of *two* operations. The tooth is thus often out, before the patient is aware of it. Being greatly delighted, he is apt to indulge in extravagant praise of the manner in which it was done, *ninifying* instead of *magnifying* his sufferings. "Why, your whole operation hurt me less than Dr. Smith did in cutting the gums," much of which is only imagination; but if we can get people to imagine themselves well served, it is all right, for *every body loves to be humbugged!*

This simplifying of the operation is especially necessary in extracting teeth for children, who will not often submit to be hurt more than once at a sitting.

But there are cases in which the *lancet must be used heroically*. These are when the teeth, either by accident or decay, have broken down to, or below the alveolar processes. The gum must then be divided to an indefinite extent, towards the apex of the fang, depending in length on its suppossed strength. A flap is then to dissected, each way from this line, sufficient in extent to allow the beaks of the forceps to slip under them and coming in contact with the processes, they must be cut, or crushed through, thus removing them with the fang. The folds, or flaps of the gum then fall together, the divided line often unites by first intention, protecting the other parts in there process of healing and absorption. To facilitate this operation the beaks of the forceps should be longer than usual, and, grasping with a firm hold, it will often happen that the pressure on the alveolus will be sufficient, with its elasticity without being either cut or broken, to *squeeze* out a fang, just as the pulp of a grape is ejected from the skin by pressure. But as a general thing, the fangs are only removed at the sacrifice of the alveolus. Nor, indeed, can they sometimes be removed at all, without subjecting the patient to more pain than seems to be justifiable. I have said the beaks of of the forceps should be longer than usual. The blade also should be longer, that more leverage power may be given. I have learned, by experience, that very long forceps are a good thing with which to break *teeth*, as well as alveolar processes. Having sometimes failed in extracting teeth, for want of physical strength, especially in the superior molars, I ordered forceps one foot in length. With these I seldom fail to—*break a tooth*! and I am not alone in this sort of success; for one day I loaned them to my distinguished friend, Dr. Driggs, who afterwards told me he suceeded admirably—in breaking off the tooth! He has not wanted to borrow them since! After I had extracted one tooth, and broken two or three, I sent them back, to have them so altered that the *extracting* should have the majority. After a time they were returned, so altered that they cut off the tooth. I then took them to a celebrated manufacturer in Philadelphia, who so changed them that they now break a tooth with almost unerring certainty! It is proper here to remark, that Dr. D. did not borrow them *till after my return from Philadelphia.*

I do not often use them in *extracting!* I try everything else first. It is sometimes a satisfaction to do *something*, even if you cannot do what you intended; but it never afforded me any particular pleasure to break a tooth—I had *rather do nothing.* I once tried three times to extract the first left superior molar, for a servant girl of some 17 or 18 summers—could not move it—patient went home. My partner returned—I told him what I had *tried to do.* He "would like to see a tooth *he could'nt extract!*" In a few days he *saw it!* With his fourth effort he broke it; destroyed the exposed pulp and sent her home! I believe if I had had my long forceps *I could have done as much!* No doubt, skill, as well as strength, is required to properly extract teeth; though it does not seem particularly essential to success, for it often happens that men who have most reputation for extracting teeth, are most miserable general operators; nor do I believe they excel in extracting, but having the happy faculty of winning the confidence of their patients, they convince them that *bad* is *good* and *pain* is *pleasure.* This is their essential element of success; for as I have said, people love to be humbugged. They will leave a man who has never made them a bad operation, nor charged them an exorbitant fee, and run to some humbug-stranger—some "celebrated" Quack,—pay him double the price for bad operations, that they have usually paid for bad ones. They then run to, and fro, up and down the earth, crying "come and see this wonderful Dentist who extracts teeth without pain, the gums are all "*healed up*" by the next day! he plugs teeth so *easy*! And talks so nicely! O he's the best dentist in the world!."

Of the various devices for extracting teeth, the forceps every where take the precedence, the Elevator, and the Key following in the wake, the latter of which, seems to be nearly abandoned *by Dentists.*

I will briefly notice a few patterns of Forceps and the mode of using them. For all teeth with single fangs, the beaks are to be made smooth on their approximal surfaces, which, when held up, shall present the surface of an imaginary frustrum of a cone, the tooth itself being the cone, or at least somewhat conical in shape. Pressure on the blades, with very little of other force or motion, will dislodge them with a popping, snapping, sound, as if the tooth were broken.

Being all nearly alike in shape, we thus dispose of all the teeth with single fangs, and proceed to notice the molars, beginning with the first inferior. The Forceps for these are to have their beaks somewhat triangular, or tri-surfaced, taper steadily to points, and so curved that in grasping the tooth between the fangs, their approximation will lift it by the pressure of the hand upon the blades; the points resting on the alveolar processes, which serve as a fulcrum for the lifting, leverage force. These forceps, if properly made, will not often either break or split the tooth.

Those for the second (molars) should have their points of adaptation—one for each fang, and one for the space between the fangs, which, on account of their proximity, do not furnish a sufficient guide for forceps with single sharp pointed beaks. More physical force in conneetion with the usual internal and external movements, is required to luxate their attachments, the forceps fitting well around the neck of the tooth. Those for the third molars—"dentes sapientiæ"—are rather for *prying* than *lifting*; the second molars forming the fulcrum, while the closing of the beaks *between* the teeth, together with a backward, upward and inward force compels them to quit their habitation. The excellence of these forceps seems to be overlooked by some Dentists. The backward curve of the fangs of these teeth, together with their wedged position, renders them difficult to extract by the application of direct force; but by putting into practice the method I have attempted to describe, the operation is very simple.

I was once in the office of a Dentist who had bought the instruments of his predecessor. I was admiring the excellence of some of his forceps, and especially those for the inferior wisdom teeth. He did not appreciate them, nor my judgement. He said "they would split the teeth." I told him how to use them. He soon changed his mind about them. I was surprised to find a man of my own age, who did not know how to use so invaluable an instrument.

We now pass to the superior molars. These usually have three fangs, the inner one standing boldly alone, at an angle of 10° to 20° serves as a firm prop, preventing the possibility of much inward motion, the outer and direct force must be increased in corresponding ratio, the outer beak of the forceps being so sharpened and fitted that it will hold firmly in the groove made by the separation of the fangs, while the inner one is to be fitted to the base of the lone fang.

Ordinary physical strength, if properly applied, is sufficient to extract these teeth—*on the right side*! But I have nothing to say about those of the left. I cannot extract them. I sometimes "pull" at them; and, and by means of main strength, I get them out. But they do not suit me. I do not like their "style." They are "*too* set in their way;" and being on the opposite side of my patient, I cannot easily get at them. I once came near getting my own *neck* broken in a fruitless effort to extract one! Leaving them for some abler operator, I pass to the second molars, which are so like the first that no description of either tooth or instrument need be given. Lastly we find the superior wisdom teeth have such insignificient little fangs that but little skill or force need be applied to them, and coming so nearly under the head of "teeth with single fangs," I refer them to that class of teeth.

There are but two positions usually taken in extracting teeth, that for all lower teeth (except the wisdom and first molar on the right side) being behind and above the patient; that for all others being rather in front, and to the right side. By common consent, everybody expects something with which to wash the mouth and gums, after extraction. Nothing but a little tepid water is really needed; but you must give them something as a placebo to quiet their fears of bleeding too much or to keep them from "taking cold," or to make their gums "*heel up*." The simplest, and probably the best thing is a little Tinct. Arnica in warm water. This will prevent their calling for "*Whiskey*," or "Salt," or

"*Camphor*," and will probably assist nature a little in performing her wonted functions. But you should always leave the door open for your patient to "take cold!" If you perform a bad operation, it is a convenient hobby on which to saddle your blunders; your teeth set on pivots never give pain, nor induce an abscess unless your patient has *taken cold!* If you treat the pulp cavity and fill a dead tooth, or fill it without any preliminary treatment, it always does well, unless your patient "*takes cold*! If you take out a portion of the pulp, leaving the rest, partially paralysed, to "die and rot," and fill the tooth over it, it always does well, unless your patient "takes cold!" I would not have you close this door! It might compel you to "tell the *truth* and shame"—*yourself.*

POST SCRIPT.—Since writing the above, in regard to superior molars on the left side, I learned to take a position not laid down in the Books, so for as my observation has extended.

You may smile, but I do not care. If I cannot extract them in a legitimate way, and upon "approved principles," I have the right to try a little strategy.

Now hold your breath, while I go round on *the other side of my patient*, where by turning his face *well from* me, and taking a position *well in front* of him, I can get at these teeth in such a way that I have more control over my own body, and can hold the head of my patient equally well with my left hand. By thus changing my *line of base*, I am enabled to operate with my elbow nearer my body, giving me better use of my own physical powers.

I thank you for kind attention.

